# A Unique Case of Exploratory Laparotomy in the Setting of Large Bowel Obstruction Led to the Detection of Pancreatic Carcinoma

**DOI:** 10.7759/cureus.21565

**Published:** 2022-01-24

**Authors:** Smriti Kochhar, Allison Zuckerberg, Mariusz Kocur, Veera Jayasree Latha Bommu, Ifeanyi Ikwuanusi, Thomas Lake, Harrison Cotler, Pramil Cheriyath

**Affiliations:** 1 Medicine, Hackensack Meridian Ocean Medical Center, Brick, USA; 2 Medicine, Rowan School of Osteopathic Medicine, Stratford, USA; 3 Internal Medicine, Hackensack Meridian Ocean Medical Center, Brick, USA; 4 Colorectal Surgery, Hackensack Meridian Ocean Medical Center, Brick, USA; 5 General Surgery, Hackensack Meridian Ocean Medical Center, Brick, USA

**Keywords:** splenic mass, metastatic pancreatic cancer, large bowel obstruction, metastasis, exploratory laparotomy, pancreatic adenocarcinoma

## Abstract

Pancreatic adenocarcinoma is the second most common gastrointestinal cancer after colon cancer. There is a delay in the detection of pancreatic adenocarcinoma as it remains asymptomatic in many individuals until it has metastasized to different parts of the body. We present a case of pancreatic cancer causing a large bowel obstruction in a 78-year-old female, detected during an exploratory laparotomy. Despite the increased incidence of pancreatic cancer, there are no screening guidelines that have been enacted for early detection and cure. Practicing clinicians should keep pancreatic cancer in the differential in high-risk individuals.

## Introduction

Pancreatic cancer accounts for about 3% of all cancers in the US and about 7% of all cancer deaths [1]. At presentation, the most frequent symptoms are asthenia, anorexia, weight loss, abdominal pain, and choluria [2]. Abdominal pain in the elderly consists of a wide range of differentials, which can be very difficult to assess in an outpatient setting. Therefore, it is important to take a complete history and physical, especially in cases of abdominal pain that is refractory to medical management. We report a rare, complicated case of a 78-year-old Caucasian female who presented with a large bowel obstruction in the setting of pancreatic carcinoma. Through this case study, we hope to bring clinical aspects that can aid in the early detection of pancreatic carcinoma to attention. Non-metastasized carcinoma has a favorable long-term prognosis and treatment, decreasing the healthcare burden [2].

## Case presentation

A 78-year-old Caucasian female with a past medical history of hypertension and arthritis presented to the hospital with a complaint of abdominal distension for 10 days prior to admission. She had not had a bowel movement or passed gas for the past six days. She complained of anorexia, nausea, vomiting, and episodic fevers. The patient had seen multiple doctors for the abdominal discomfort and was diagnosed with bloating and diverticulitis. Because of the worsening of her symptoms, she decided to come to the emergency department. Her vitals in the emergency department were as follows: blood pressure 136/64 mmHg, pulse 95 beats per minute, respiratory rate 20 breaths per minute, saturating 95% oxygen on room air, temperature 98.0 ℉. Physical exam was unremarkable other than the abdominal distension and the right upper quadrant mass. Laboratory revealed white blood cell (WBC) count 6.7 k/µL (4.5-11), hemoglobin 12.2 g/dL (12-16), platelets 462 10*3/µL (140-450 10^3^/µL), serum creatinine 0.94 mg/dL (0.44-1.00), and sodium 131 mmol/L (136-145), chloride 94 mmol/L (96-110 mmol/L), glomerular filtration rate (GFR) 58 mL/min/1.73 m^2^ (>60 mL/min/1.73 m^2^). The patient was reported to be a lifetime non-smoker and said that she drinks alcohol occasionally. Additionally, she was reported to have a family history of lung cancer in her mother, head and neck cancer in her son, and one niece with pancreatic cancer. Her prior colonoscopy in 2019 was unremarkable.

Imaging studies

**Figure 1:**
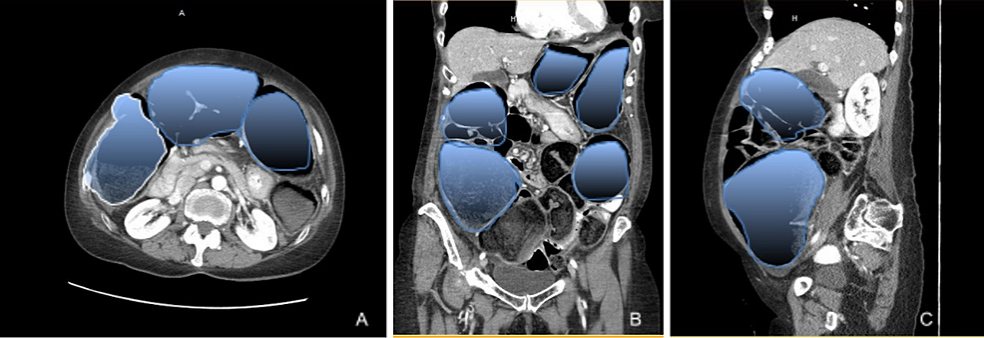
Non-contrast computed tomography axial view (A), coronal view (B), and sagittal view (C) of the abdomen showing dilated loops of large bowel within the abdominal cavity (area highlighted).

Computed tomography (CT) of the abdomen with contrast showed gas and fluid distended large bowel loops with a distended cecum measuring up to 12.5 cm in diameter. The large bowel loops were dilated up to the splenic flexure (Figure 1), and a mass-like lesion was also identified there, measuring 3.3 cm × 3.3 cm with surrounding fat stranding. This lesion was adjacent to the spleen. Heterogeneous density identified at the spleen was suspicious for lesion/tumor extension into the spleen. The large bowel loops distal to this lesion appeared collapsed.

**Figure 2:**
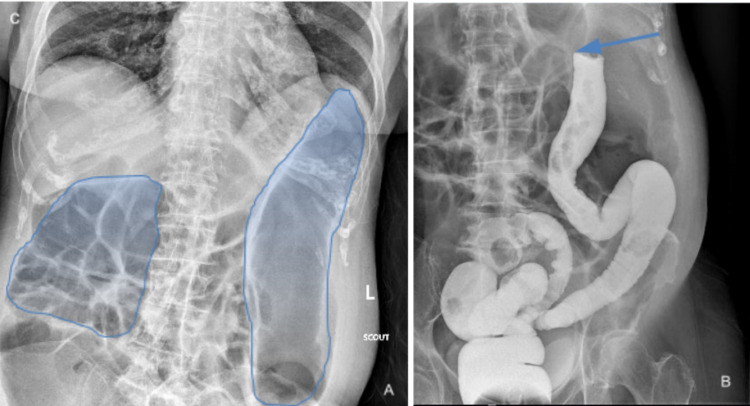
X-ray of the large intestine showing dilated loops of bowel with loss of haustra in descending colon as highlighted in image (A). Gastrograffin enema in image (B) shows splenic flexure obstruction (blue arrow).

Given these findings, the patient also received an ​X-ray contrast enema with single contrast, which showed ​​opacification of the rectum, sigmoid colon, and descending colon, probably with a cutoff contrast at the proximal descending colon compatible with an obstructing mass (Figure 2). With the concern of large bowel obstruction due to a splenic mass, the patient was taken to the operating room for an exploratory laparotomy.

**Figure 3:**
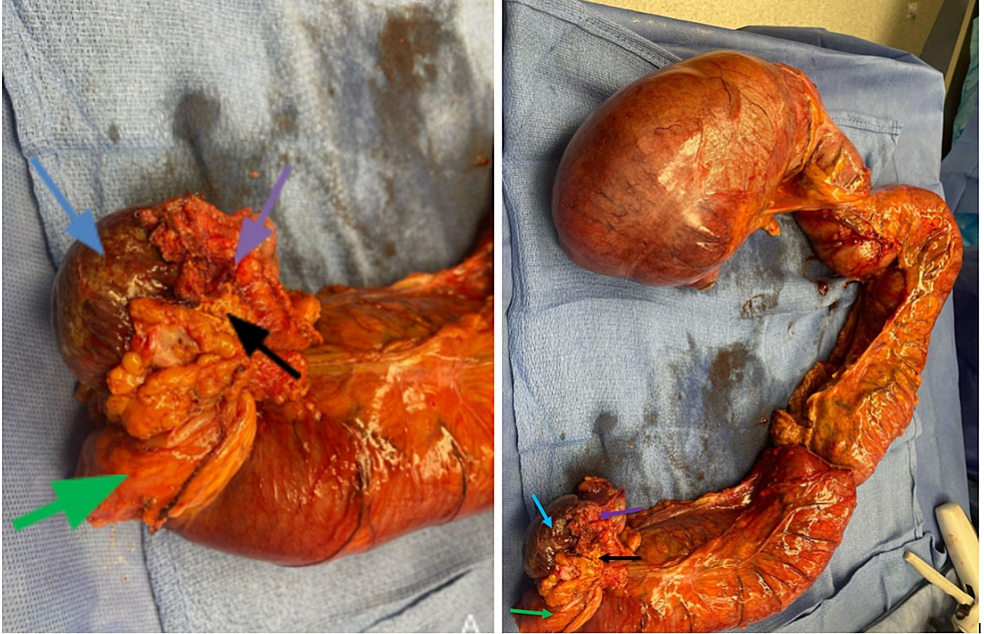
Gross anatomy in image (A) of spleen (blue arrow), distal pancreas (purple arrow), left adrenal (black arrow), greater curvature of stomach (green arrow) which were removed along with the dilated loops of large bowel as seen in image (B).

During the laparotomy, the patient had a very distended colon. There was an impending perforation of the ascending colon, and there was a splitting of the taenia coli. On further dissection distally in the lesser sac, it was evident that the tumor at the splenic flexure was invading the spleen, stomach, and possibly the distal pancreas. Therefore, in order to provide relief from obstruction, she underwent en bloc spleen, distal pancreas, left adrenal, greater curvature of the stomach (Figure 3), total colectomy with ileo-rectal anastomosis, and closure of the skin with two Jackson Pratt (JP) drains were left in the peritoneal cavity. The pathology of the specimens showed ​​adenocarcinoma, favoring pancreatic ductal carcinoma involving the pancreatic tail, splenic hilum and parenchyma, gastric and colonic wall, adrenal capsule, and surrounding fat. The tumor also involved two adjacent nodes (one peripancreatic and one pericolic) out of a total of 15 lymph nodes. This resulted in the staging at PT3N1.

Post-operative labs were checked for tumor markers carcinoembryonic antigen (CEA) which was normal at 1.2 but the CA 19-9 and CA-125 were elevated at 203 U/mL (<34 U/mL) and 104 U/mL (<35 U/mL), respectively. Lactate dehydrogenase (LDH) and lactic acid were also elevated at 217 U/L and 5.1 (0.5-2.0 mmol/L). Her diet was changed to total parenteral nutrition with everyday monitoring of her electrolytes and sugars that ranged from 38 to 224 (70-99 mg/dL). Post-op was complicated by lethargy and desaturation for which the patient was reopened 12 days after the initial surgery for laparotomy where lysis of adhesions, small bowel resection, partial colectomy, loop ileostomy, partial gastrectomy, and pancreatic duct fistula repair were performed. The patient improved clinically thereafter and was eventually discharged for acute rehabilitation nine days post-second surgery.

## Discussion

The pancreas is a part-intraperitoneal, part-retroperitoneal organ that secretes enzymes to digest food, namely fats. It is made up of some endocrine cells and mostly exocrine cells, which include glands and ducts [3]. Of pancreatic cancers, the most common type is exocrine, and 95% are adenocarcinomas. These usually develop in the ducts of the pancreas and, less often, they develop from acinar cells, which make pancreatic enzymes [3]. Endocrine pancreatic cancers may also occur. However, these are less common and associated with different risk factors, causes, signs, and symptoms [3]. Additionally, they are tested and treated differently with different prognoses, so they are not discussed in this case report.

Weight loss (90%), abdominal pain (75%), painless jaundice (70%), anorexia (64%), pruritus, palpable, non-tender distended gallbladder, dark urine, and acholic stools are common presenting features of pancreatic adenocarcinoma [4]. Metastatic pancreatic cancer presenting as a large bowel obstruction has only been described on a case-by-case basis. In 1981, the first known case was described in an individual whose pancreatic cancer directly infiltrated the transverse colon [5]. In 2012, a patient who presented with abdominal pain was found to have a pancreatic tail mass on CT, and three days later, he developed large bowel obstruction secondary to a mass in the descending colon, which resulted in emergency distal pancreatectomy, left hemicolectomy, partial adrenalectomy, and lymph node dissection [6]. With no recurrence two years later, the authors called for aggressive surgical management as the approach to such cancers. This patient was found to have poorly differentiated anaplastic pancreatic cancer [6]. However, as evidenced in our patient, pancreatic adenocarcinoma also has the potential to spread and present similarly and emergently. Our patient also showed splenic atrophy, which we hypothesize is due to compression and invasion of the splenic artery by the pancreatic tumor. This is also a poor indicator of survival and classifies the tumor as T4 [7]. Additionally, in 2016, another case of large bowel obstruction secondary to metastatic pancreatic cancer was reported [8]. Similar to our patient, obstruction was the presenting symptom and the patient was taken immediately to an exploratory laparotomy. Metastatic pancreatic cancer was diagnosed after resection and final pathology of the obstructing colonic mass as well as an intraperitoneal mass [8].

Initially, our patient’s symptoms were thought to be due to undiagnosed colorectal cancer. Large bowel obstruction is most commonly caused by malignancy, volvulus, and stricture, and of these, colorectal carcinoma is the most common cause [7]. Up to 20% of patients with colorectal carcinoma may be diagnosed at the time of an emergency presentation [5]. Improvements in the screening, diagnosis, and treatment of colorectal cancer since the 1980s have resulted in fewer patients presenting with emergency presentations (such as with obstruction) [9]. However, this was found to have increased again since March 2020 due to the COVID-19 pandemic [10]. In cases such as our patient, a large bowel obstruction may be the presentation of metastatic malignancy such as pancreatic cancer. Further, the pandemic may be leading to individuals presenting with more advanced courses than in the past.

According to the American Cancer Society, pancreatic cancer accounts for around 3% of all malignancies and about 7% of all cancer deaths in the United States [11]. Unfortunately, pancreatic cancer is the third most common cause of cancer death and is projected to become the second leading cause of cancer death by the year 2030 [12]. Pancreatic cancer may be located in the head, body, or tail and its location determines presenting signs and symptoms. Pancreatic cancer in the head is associated with jaundice and obstructive symptoms [8]. However, body or tail cancer may not have symptoms until metastasis, when the prognosis is worse. In general, pancreatic cancer is most commonly diagnosed at an advanced stage because of the late or insidious onset of clinical features. It is estimated that 52% of patients already have metastasized disease at the time of diagnosis [1]. Early surgical resection is associated with improved mortality and morbidity; however, at metastasis, treatment is often palliative as surgical resection may not be possible. Five-year survival depends on the extent, spread, and resectability of the tumor. However, it is generally poor, with its average five-year survival being 10.8% from 2011 to 2017. If distant metastasis is present at the time of diagnosis, relative survival is even lower at 5% [1].

At present, the American Gastrointestinal Association advises against screening for individuals at average risk for pancreatic cancer [13]. Similarly, the US Preventive Services Task Force recommends against screening for asymptomatic adults (Grade D recommendation) [12]. Features that may deem an individual high risk include having first-degree relatives with pancreatic cancer with at least two genetically related relatives or genetic syndromes associated with an increased risk of pancreatic cancer [13]. Poorer outcomes of pancreatic cancer is because it often presents at an advanced stage [14]. Given this, the medical community and patients are in need of a screening tool that would allow for earlier intervention that would improve mortality and morbidity.

## Conclusions

Usual suspects of large bowel obstruction include colorectal carcinoma, volvulus, adhesions, and strictures from diverticulitis and hernias. Pancreatic adenocarcinoma can lead to invasion into the colon, spleen, adrenals, and other surrounding organs but also distant metastasis spread hematogenously. The most common site of metastasis is the liver. Physicians should be aware of the deadly nature of large bowel obstructions, which can be a pancreatic cancer invasion, especially if the splenic flexure is involved.
